# Prognostic value of simultaneous ^18^F-FDG PET/MRI using a combination of metabolo-volumetric parameters and apparent diffusion coefficient in treated head and neck cancer

**DOI:** 10.1186/s13550-018-0357-9

**Published:** 2018-01-10

**Authors:** Yong-il Kim, Gi Jeong Cheon, Seo Young Kang, Jin Chul Paeng, Keon Wook Kang, Dong Soo Lee, June-Key Chung

**Affiliations:** 1 Department of Nuclear Medicine, CHA Bundang Medical Center, CHA University, Seongnam, Republic of Korea; 2 Department of Nuclear Medicine, Seoul National University Hospital, Seoul, Republic of Korea; 3 Department of Nuclear Medicine, Asan Medical Center, University of Ulsan College of Medicine, Seoul, Republic of Korea; 4 Department of Nuclear Medicine, Seoul National University College of Medicine, 101 Daehak-ro, Chongno-gu, Seoul, 03080 Korea

**Keywords:** Head and neck cancer, Integrated PET/MRI, Metabolic tumor volume, Total lesion glycolysis, Apparent diffusion coefficient

## Abstract

**Background:**

The aim of this study was to determine the usefulness of combined positron emission tomography (PET)/magnetic resonance imaging (MRI) parameters provided by simultaneous ^18^F-fluorodeoxyglucose (FDG) PET/MRI for the prediction of treatment failure in surgically resected head and neck cancer. We hypothesized that PET parameters corrected by tumor cellularity (combined PET/MRI parameters) could predict the prognosis. On regional PET, maximum standardized uptake value (SUVmax) was measured as metabolic parameters. In addition, metabolic tumor volume (MTV) and total lesion glycolysis (TLG) were checked as metabolo-volumetric parameters. Mean apparent diffusion coefficient (ADCmean) of tumor was evaluated as the MRI parameter on the ADC map. Ratios between metabolic/metabolo-volumetric parameters and ADC were calculated as combined PET/MRI parameters. PET, MRI, and combined PET/MRI parameters were compared with clinicopathologic parameters in terms of treatment failure.

**Results:**

Seventy-two patients (mean age = 55.9 ± 14.6 year, M: F = 45: 27) who underwent simultaneous ^18^F-FDG PET/MRI before head and neck cancer surgery were retrospectively enrolled. Twenty-two patients (30.6%) showed tumor treatment failure after head and neck cancer surgery (mean treatment failure = 13.0 ± 7.0 months). In the univariate analysis, MTV (*P* = 0.044) and ratios between metabolo-volumetric parameters and ADC (MTV/ADCmean, *P* = 0.022; TLG/ADCmean, *P* = 0.044) demonstrated significance among ^18^F-FDG PET/MRI parameters. Lymphatic invasion (*P* = 0.044) and perineural invasion (*P* = 0.046) revealed significance among clinicopathologic parameters. In the multivariate analysis, MTV (*P* = 0.026), MTV/ADCmean (*P* = 0.011), and TLG/ADCmean (*P* = 0.002) with lymphatic invasion (*P* = 0.026, 0.026, and 0.044, respectively) showed significance.

**Conclusions:**

Combined PET/MRI parameters (PET metabolo-volumetric parameters corrected by tumor cellularity) could be effective predictors of tumor treatment failure after head and neck cancer surgery in addition to MTV and clinicopathologic parameter.

**Electronic supplementary material:**

The online version of this article (10.1186/s13550-018-0357-9) contains supplementary material, which is available to authorized users.

## Background

Head and neck cancer involves many structures, including the nasal cavity, oral cavity, tongue, pharynx, larynx, and salivary glands, and the 5-year survival rate is about 65% [1]. ^18^F-fluorodeoxyglucose (FDG) positron emission tomography (PET)/computed tomography (CT) is widely used in current clinical practice for cancers. Regarding head and neck cancer, ^18^F-FDG PET/CT is recommended in diagnosis, staging, and recurrence detection [2]. Furthermore, ^18^F-FDG PET/CT has been reported to be effective in predicting head and neck cancer recurrence using quantitative parameters [3].

Simultaneous PET/magnetic resonance imaging (MRI) is a recently developed technology that is expected to be a better imaging modality than each modality alone due to the complementary information of each modality [4, 5]. In addition, simultaneous PET/MRI is expected to be more valuable than PET/CT because it involves less radiation exposure and offers better soft-tissue contrast resolution [6]. In head and neck cancer, MRI has an important role due to its excellent soft-tissue contrast, which provides anatomy of small structures in detail [7]. A previous study reported that simultaneous PET/MRI is feasible for the staging of head and neck cancer and the discordant result of PET and MRI might have a synergistic effect for accurate staging [8].

The main advantage of simultaneous PET/MRI is that several functional imaging types can be performed by MRI [9]. Among them, diffusion-weighted imaging (DWI), which enables the assessment of the random (Brownian) motion of water molecules without the injection of contrast materials or radiotracers, has been studied [10]. DWI can be quantified by the apparent diffusion coefficient (ADC), which reflects the cellularity of tumors [11]. In reference to head and neck cancer, ADC has been shown to be effective in diagnosis, staging, and therapeutic effect evaluation [12, 13]. In addition, ADC could be useful for predicting treatment response in head and neck cancer [14]. However, DWI using ADC has yet to be transferred to the clinical domain.

We hypothesized that PET parameters of simultaneous ^18^F-FDG PET/MRI could be more accurately characterized to reflect the aggressiveness of tumors by correcting tumor cellularity utilizing ADC in surgically resected head and neck cancer. We evaluated prognostic value of metabolic and metabolo-volumetric parameters of PET, ADC of MRI, and combined PET/MRI parameters in addition to several clinicopathologic parameters and compared them.

## Methods

### Patients

From March 2013 to December 2015, 128 patients underwent simultaneous ^18^F-FDG PET/MRI as a preoperative workup for head and neck cancer in our institution. Among them, patients who met the following inclusion criteria were retrospectively enrolled in this study: (1) underwent simultaneous ^18^F-FDG PET/MRI less than 2 months before surgery, (2) no evidence of distant metastasis during preoperative workup, and (3) follow-up more than 12 months after surgery in case of no treatment failure. Patients who performed initial treatment as concurrent chemoradiation therapy (CCRT) were excluded. Patients were routinely checked using laryngoscopy and imaging studies, such as ultrasonography, CT, or MRI, every 3–4 months in the first year after surgery and every 6 months thereafter. Additional imaging studies were performed when serum carcinoembryonic antigen (CEA) (checked at routine checkups at 3–4 months intervals; threshold of 5 ng/ml) was increased, or other suspicious symptoms or signs were presented. Treatment failure was mainly confirmed by histopathology when recurrence or metastasis was suspected during follow-up of laryngoscopy and imaging studies. When the hisopathologic confirmation was not feasible, radiologic confirmation was made based on mutual decision by radiologists and oncologists. The study design and waive of informed consents were approved by the Institutional Review Board (IRB) of our institution.

### Protocols of simultaneous ^18^F-FDG PET/MRI

After fasting for at least 6 h, patients were injected with 0.14 mCi/kg of ^18^F-FDG. Serum glucose levels were obtained before ^18^F-FDG injection, and levels were less than 200 mg/dl in all patients. After 50 min of ^18^F-FDG administration, simultaneous PET/MRI scanning was performed using an integrated PET/MRI scanner (Biograph mMR, Siemens Healthcare, Erlangen, Germany). Whole-body and regional PET/MRI images were sequentially obtained, and magnetic resonance (MR) examinations were performed with a 3T MR imaging unit.

First, whole-body PET imaging was performed from the head to the distal thigh with an acquisition time of 3 min/bed and axial field of view (FoV) of 25.7 cm. Dixon-VIBE MRI was simultaneously acquired in the axial orientation to correct the PET attenuation before injection of gadolinium (Dotarem, Guerbet, France; slice thickness of 7 mm). After whole-body PET/MRI imaging was performed, the patient underwent regional PET imaging of the head and neck with an acquisition time of 10 min and regional MRI, including DWI at the same time. DWI (sequence of spin-echo echo-planar imaging (SE-EPI) with mode of strong fat saturation) was performed in the transverse plane before the contrast material injection with *b* values of 0 and 1000 /mm^2^. The pulse sequences of DWI were defined as follows: repetition time (TR) 9600 ms, echo time (TE) 93 ms, FoV 240 mm, slice thickness 4 mm, slice gap 10 mm, and voxel size 1.1 × 1.1 × 4.0 mm. None of the patients was excluded due to artifacts of the DWI sequence. Following the injection of gadolinium, a T1-weighted sequence was acquired in the axial, coronal, and sagittal directions. Axial images of regional PET and MRI were parallel to the anterior and posterior commissure line of the brain. Lutetium oxyorthosilicate (LSO)-based avalanche photodiodes were used for PET image acquisition. Images were corrected for attenuation and reconstructed on a 172 × 172 matrix using a Gaussian filter with a 6.0-mm full width at half maximum and a three-dimensional ordered-subsets expectation maximization (OSEM) algorithm (three iterations, 21 subsets, zoom of 1.0).

### Image analysis of simultaneous ^18^F-FDG PET/MRI

**Fig. 1:**
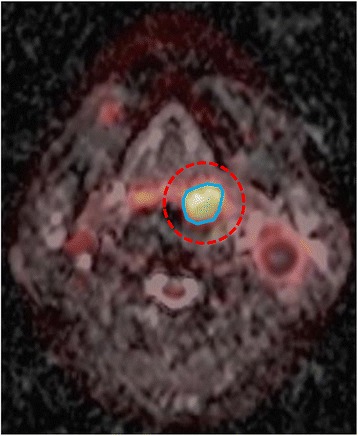
Measuring methods of simultaneous ^18^F-FDG PET/MRI parameters. As an initial step, the regional PET and ADC map were integrated. For the acquisition of PET parameters (SUVmax, MTV, and TLG), a spherical VOI (dashed red circle) was drawn to include the whole tumor. An isoactivity contour (solid blue circle) was automatically drawn by setting a threshold of SUV 2.5, and PET parameters were acquired. For an MRI parameter, a VOI was drawn along the isoactivity contour of PET, and the ADCmean of the tumor was evaluated

All of the reviews of PET images and determination of PET parameters were performed using syngo.via software (Version VA11A; Siemens Healthcare, Erlangen, Germany) with a setting that allowed maximum intensity projection (MIP) and three-dimensional displays (transaxial, coronal, and sagittal) of PET, MRI, and fused PET/MRI images. As a first step, regional PET and diffusion-weighted MRI were fused, and we drew a large volume-of-interest (VOI) to include whole head and neck cancer. PET and MRI parameters were checked by setting isoactivity contours, and combined PET/MRI parameters were calculated (Fig. 1).

For the acquisition of PET parameters, the standardized uptake value (SUV) by body weight was measured for quantitative analysis according to the following equation: SUV = (tissue radioactivity (Bq)/tissue weight (g))/(total injected activity (Bq)/body weight (g)). As metabolic parameters of PET, maximum SUV (SUVmax) was measured by the highest pixel uptake. As metabolo-volumetric parameters of PET, an isoactivity contour was drawn by setting a threshold of SUV 2.5, the most frequently used threshold in previous studies [15, 16]. Metabolic tumor volume (MTV) was measured automatically by the software, and total lesion glycolysis (TLG) was calculated by multiplying SUVmean by MTV. As an MRI parameter, mean ADC (ADCmean) for head and neck cancer was measured by manually drawing a VOI on the ADC map using monoexponential ADC calcucalation method. The VOI was drawn along the isoactivity contour of PET [17, 18]. Combined PET/MRI parameters (PET parameters corrected by tumor cellularity) were calculated as the ratio between metabolic/metabolo-volumetric PET parameters and ADC. The measurement of PET and MRI parameters was done by two nuclear medicine physicians (YK and SYK; 9 and 5 years of experience) twice and averaged in blinded state to clinical data and without knowledge of histology.

### Clinicopathologic parameters

Clinicopathologic parameters were obtained from a medical record review. Age, tumor site, and adjuvant therapy were checked as clinical parameters. Human papillomavirus (HPV) status, T stage, N stage, TNM stage, lymphatic invasion (lymph vessel invasion), venous invasion, and perineural invasion on surgical tumor specimen were evaluated as pathologic parameters.

### Statistical analysis

Continuous parameters were expressed as mean ± standard deviation (SD), and categorical parameters were expressed as number. First, clinicopathologic and simultaneous ^18^F-FDG PET/MRI parameters were compared between treatment failure, and no evidence of disease groups using chi-square tests for categorical data (Mann-Whitney test for non-parametric data) and independent-samples *t* tests for continuous data. Second, the median values of simultaneous ^18^F-FDG PET/MRI parameters were identified to determine the cutoff value. Third, a Kaplan–Meier analysis and a log-rank test were done to confirm disease-free survival (DFS) and significance of simultaneous ^18^F-FDG PET/MRI parameters. Finally, univariate and multivariate Cox-regression analysis was performed to assess the effect of significant parameters. Bonferroni correction was done in Cox-regression analysis (univariate analysis) to counteract the problem of multiple comparisons. A log-log survival plot was used to identify the proportional hazard assumption. A *P* < 0.05 was considered statistically significant. All statistical analyses were performed using SPSS software (Version 18.0; SPSS Inc., Chicago, IL, USA) and MedCalc (Version 12.2; MedCalc Inc., Mariakerke, Belgium).

## Results

### Patients

A total of 72 patients were included in our study (M:F = 45: 27, mean age = 55.9 ± 14.6 year). Tumor locations were as follows: oral cavity and tongue 51.4%, pharynx 16.7%, larynx 8.3%, nasal cavity and paranasal sinuses 11.1%, and salivary glands 12.5%. TNM stages of the tumors were as follows: stage I = 23.6%, stage II = 19.4%, stage III = 26.4%, and stage IVA = 30.6%. Most of the patients performed neck dissection including surgery (60/72 patients, 83.3%), and rest of the patients (12/72 patients, 16.7%) performed tumorectomy due to early cancer. After surgical resection of the tumors, adjuvant therapy was done in 54.2% of patients (radiation therapy (RT) 33.3% and CCRT 20.9%), and mean follow-up was 32.8 ± 10.8 months. Twenty-two patients (30.6%) demonstrated treatment failure (confirmed 16 patients by histopathology, 6 patients by radiology), and mean treatment failure was 13.0 ± 7.0 months after surgery (Table 1).

**Table 1:** Baseline characteristics of patients

Characteristics	Values
Number of patients	72
Gender	M:F = 45: 27
Age (year)	55.9 ± 14.6 (range = 20–86)
Tumor site	R:L = 32: 40
Tumor location	
Oral cavity and tongue	37 (51.4%)
Pharynx	12 (16.7%)
Larynx	6 (8.3%)
Nasal cavity and paranasal sinuses	8 (11.1%)
Salivary glands	9 (12.5%)
HPV status	
Positive	10 (13.9%)
Negative	21 (29.2%)
N/A	41 (57.7%)
Follow-up after surgery (months)	32.8 ± 10.8 (range = 12–55)
Recurrence	22 (30.6%)
Recurrence after surgery (months)	13.0 ± 7.0 (range = 5–33)
Type of treatment failure	
Loco-regional recurrence	10 (13.9%)
Distant metastasis	12 (16.7%)
T stage	
T1/T2	24 (33.3%)/23 (31.9%)
T3/T4a	20 (27.8%)/5 (7.0%)
N stage	
N0	42 (58.3%)
N1/N2	12 (16.7%)/18 (25.0%)
TNM stage	
I/II	17 (23.6%)/14 (19.4%)
III/IVA	19 (26.4%)/22 (30.6%)
Adjuvant therapy	
No	33 (45.8%)
RT/CCRT	24 (33.3%)/15 (20.9%)

*HPV* human papillomavirus, *RT* radiation therapy, *CCRT* concurrent chemoradiation therapy, *N/A* not assessed

### Clinicopathologic parameters and treatment failure

Among the clinicopathologic parameters, T stage (*P* = 0.019), lymphatic invasion (*P* = 0.005), venous invasion (*P* = 0.012), and perineural invasion (*P* = 0.008) demonstrated significant results between treatment failure and no evidence of disease groups. However, age, tumor site, HPV status, N stage, TNM stage, and adjuvant therapy revealed no significant results (Table 2).

**Table 2:** Comparison of clinicopathologic parameters according to head and neck cancer treatment failure after surgery

Parameters	Treatment failure (*n* = 22)	No evidence of disease (*n* = 50)	*P* value
Age (year)	55.7 ± 16.0	56.0 ± 14.1	0.947
Tumor site (R:L)	8:14	24:26	0.360
HPV status			0.381
Positive	4	6	
Negative	4	17	
T stage			0.019*
1/2	10	37	
3/4	12	13	
N stage			0.341
0	11	31	
1/2	11	19	
TNM stage			0.201
I/II	7	24	
III/IVA	15	26	
Lymphatic invasion			0.005*
No	11	41	
Yes	11	9	
Venous invasion			0.012*
No	16	47	
Yes	6	3	
Perineural invasion			0.008*
No	12	42	
Yes	10	8	
Adjuvant therapy			0.113
No	7	26	
RT/CCRT	15	24	

*Statistically significant (*P* < 0.05)

### Simultaneous ^18^F-FDG PET/MRI parameters and treatment failure

Among the simultaneous ^18^F-FDG PET/MRI parameters, MTV (*P* = 0.049), MTV/ADCmean (*P* = 0.018), and TLG/ADCmean (*P* = 0.025) revealed significance between treatment failure and no evidence of disease groups. However, SUVmax, TLG, ADCmean, and SUVmax/ADCmean showed no significant results (Table 3). In addition, no significant differences were found between loco-regional recurrence and distant metastasis groups (Additional file 1: Table S1).

**Table 3:** Comparison of ^18^F-FDG PET/MRI parameters according to head and neck cancer treatment failure after surgery

^18^F-FDG PET/MRI parameters	Treatment failure (*n* = 22)	No evidence of disease (*n* = 50)	*P* value
PET parameters			
SUVmax	9.7 ± 3.9	9.7 ± 5.0	0.582
MTV	13.5 ± 13.3	7.5 ± 7.9	0.049*
TLG	71.8 ± 81.5	40.2 ± 52.6	0.057
MRI parameters			
ADCmean	827.3 ± 232.7	972.1 ± 365.1	0.074
Combined PET/MRI parameters			
(SUVmax/ADCmean) × 1000	12.8 ± 6.0	11.3 ± 6.7	0.208
(MTV/ADCmean) × 1000	17.8 ± 20.1	7.6 ± 7.3	0.018*
(TLG/ADCmean) × 1000	91.1 ± 102.9	40.0 ± 48.6	0.025*

*SUVmax* maximum standardized uptake value, *MTV* metabolic tumor volume, *TLG* total lesion glycolysis, *ADCmean* mean apparent diffusion coefficient

*Statistically significant (*P* < 0.05)

### Univariate and multivariate analysis

**Fig. 2:**
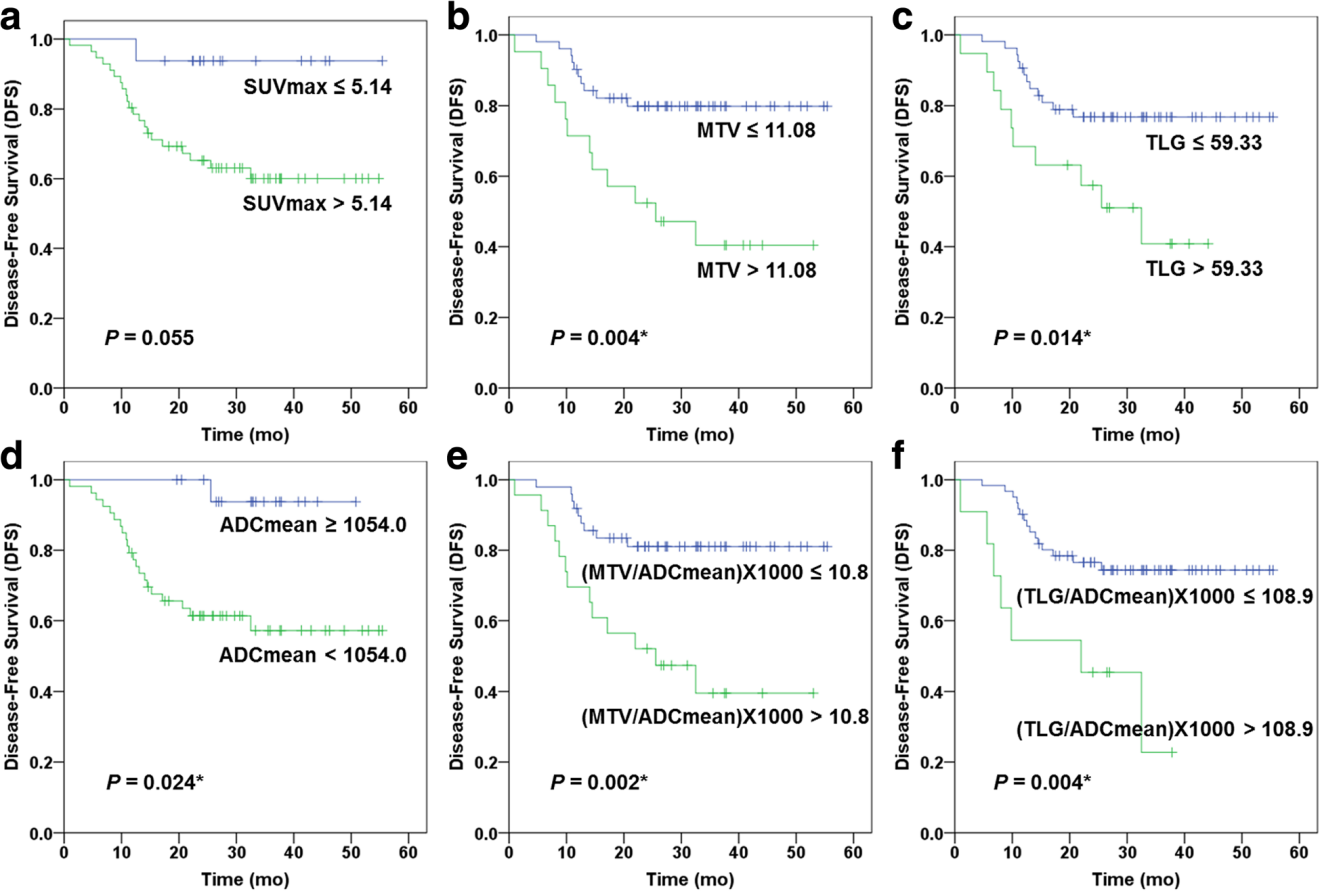
Kaplan–Meier analysis of ^18^F-FDG PET/MRI parameters for prediction of tumor treatment failure after head and neck cancer surgery. DFS and *P* values were as follows: **a** SUVmax (3-year DFS = 93.8 vs. 60.1%, *P* = 0.055), **b** MTV (3-year DFS = 79.8 vs. 40.4%, *P* = 0.004), **c** TLG (3-year DFS = 76.8 vs. 40.8%, *P* = 0.014), **d** ADCmean (3-year DFS = 93.8 vs. 57.3%, *P* = 0.024), **e** MTV/ADCmean (3-year DFS = 81.1 vs. 39.5%, *P* = 0.002), and **f** TLG/ADCmean (3-year DFS = 74.4 vs. 22.7%; *P* = 0.004)

The univariate analysis of significant clinicopathologic parameters revealed that lymphatic invasion (*P* = 0.044) and perineural invasion (*P* = 0.046) were significant parameters. Among the ^18^F-FDG PET/MRI parameters, MTV (*P* = 0.044), MTV/ADCmean (*P* = 0.022), and TLG/ADCmean (*P* = 0.044) revealed significance after Bonferroni correction. No statistical significance was found in T stage, venous invasion, SUVmax, ADCmean, and SUVmax/ADCmean (Table 4 and Fig. 2).

**Table 4:** Univariate analysis with clinicopathologic and ^18^F-FDG PET/MRI parameters

Parameters	Hazards ratio (95% CI)	*P* value	Corrected *P* value†
T stage (3/4a vs. 1/2)	2.61 (1.12–6.05)	0.026*	0.286
Lymphatic invasion (yes vs. no)	3.40 (1.47–7.85)	0.004*	0.044*
Venous invasion (yes vs. no)	3.21 (1.26–8.22)	0.015*	0.165
Perineural invasion (yes vs. no)	3.34 (1.44–7.76)	0.004*	0.044*
SUVmax (> 5.14 vs. ≤ 5.14)	2.13 (0.96–3.03)	0.055	0.605
MTV (> 11.08 vs. ≤ 11.08)	3.48 (1.50–8.05)	0.004*	0.044*
TLG (> 59.33 vs. ≤ 59.33)	2.86 (1.23–6.63)	0.014*	0.154
ADCmean (< 1054.0 vs. ≥ 1054.0)	2.02 (1.15–4.68)	0.024*	0.264
(SUVmax/ADCmean) × 1000 (> 13.9 vs. ≤ 13.9)	2.43 (1.04–5.71)	0.041*	0.451
(MTV/ADCmean) × 1000 (> 10.8 vs. ≤ 10.8)	3.82 (1.63–8.94)	0.002*	0.022*
(TLG/ADCmean) × 1000 (> 108.9 vs. ≤ 108.9)	3.76 (1.52–9.25)	0.004*	0.044*

*Statistically significant (*P* < 0.05). †Multiple comparison correction by Bonferroni

**Fig. 3:**
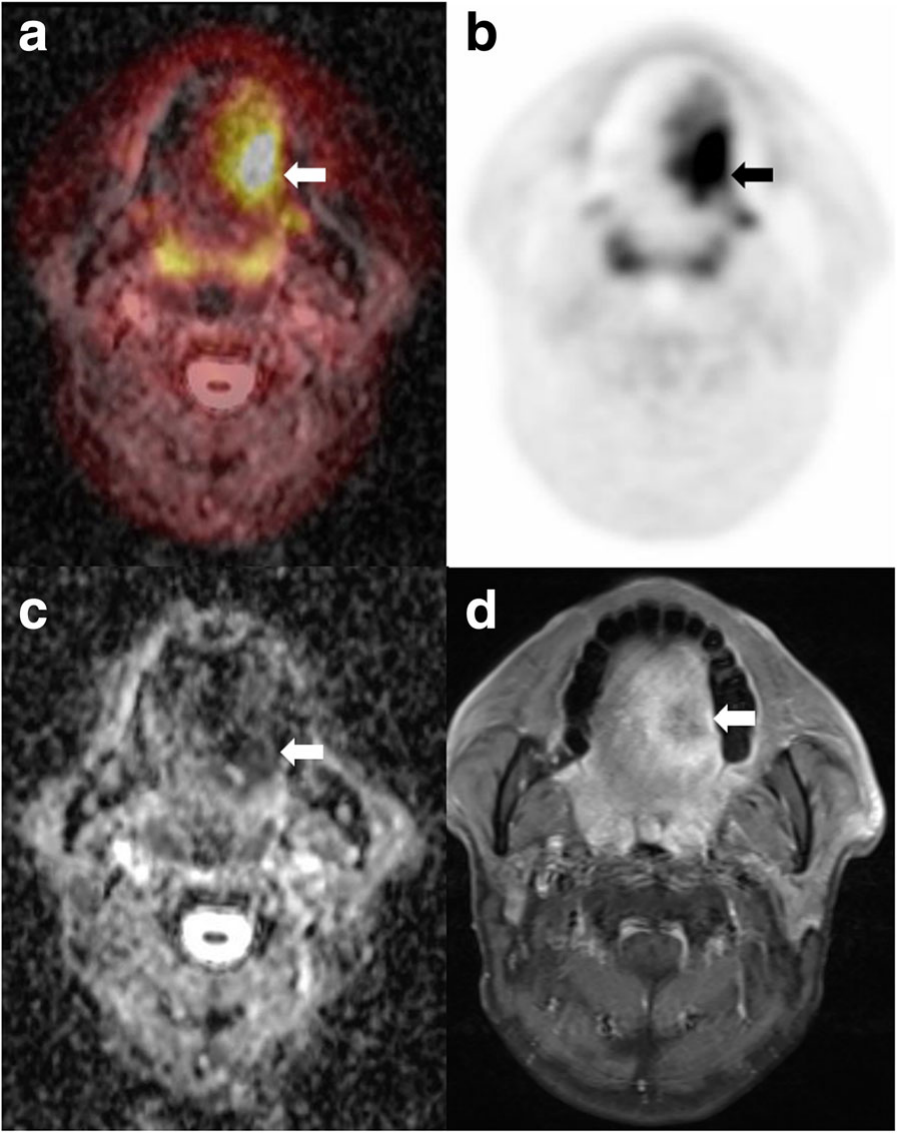
Case of tongue cancer treatment failure prediction by both MTV and combined PET/MRI parameters. A 50-year-old male underwent preoperative simultaneous ^18^F-FDG PET/MRI due to a 3.8-cm-sized left tongue cancer. **a** Fusion image of regional PET with ADC map and **b** regional PET images showed left tongue mass with hypermetabolism (arrows). **c** ADC map image demonstrated left tongue mass with diffusion restriction (arrow). **d** Gadolinium-enhanced T1 axial image revealed mass with peripheral enhancement (arrow). SUVmax (9.73), MTV (35.02), TLG (135.18), ADCmean (424.15), (SUVmax/ADCmean) × 1000 (10.37), (MTV/ADCmean) × 1000 (82.57), and (TLG/ADCmean) × 1000 (318.70) predicted tumor treatment failure. The patient showed tumor treatment failure in pleura, multiple bones, and lymph nodes 6 months after tongue cancer surgery

**Fig. 4:**
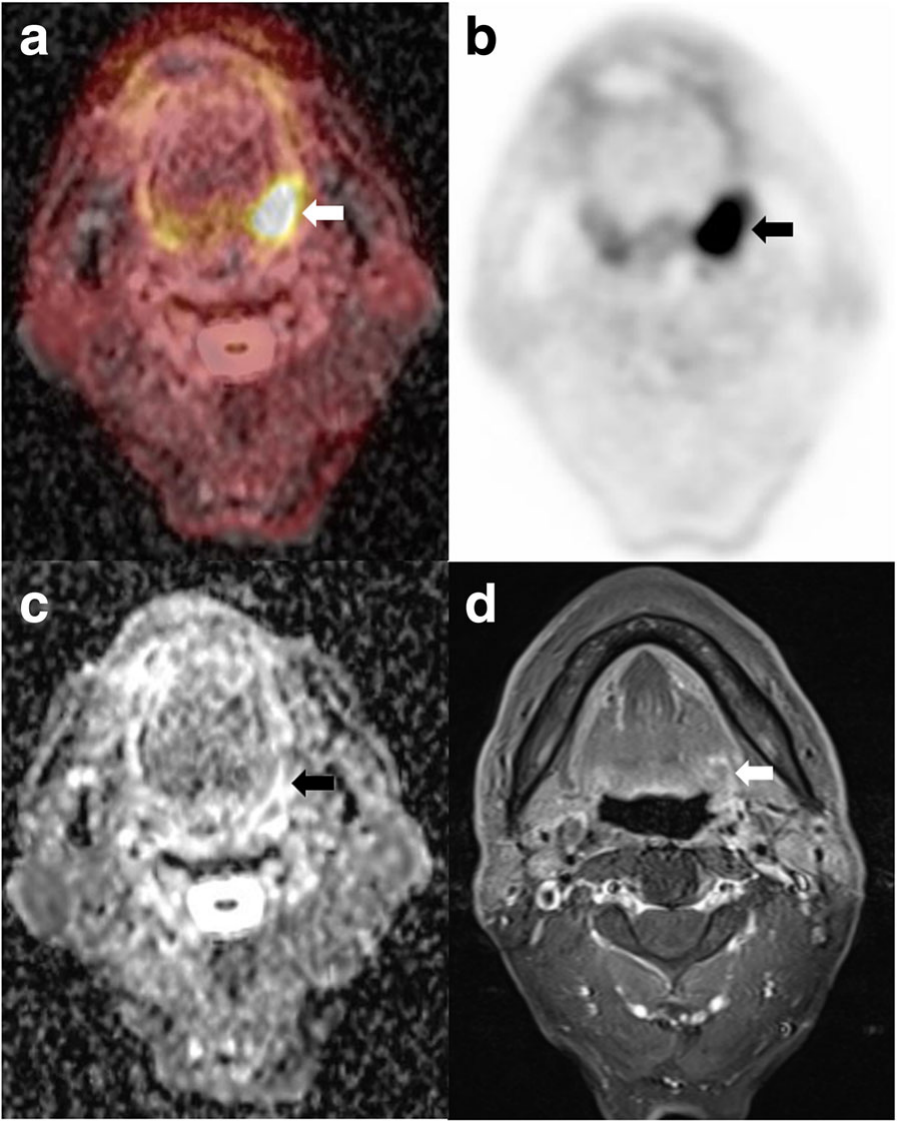
Case of tonsillar cancer with no evidence of disease predicted by combined PET/MRI parameters but treatment failure predicted by PET parameters. A 66-year-old male underwent preoperative simultaneous ^18^F-FDG PET/MRI due to a 3.8-cm-sized left tonsillar cancer. **a** Fusion image of regional PET with ADC map and **b** regional PET images showed left tonsillar mass with hypermetabolism (arrows). **c** ADC map image demonstrated left tonsillar mass with diffusion restriction (arrow). **d** Gadolinium-enhanced T1 axial image revealed mass with peripheral enhancement (arrow). ADCmean (1443.85), (SUVmax/ADCmean) × 1000 (10.37), (MTV/ADCmean) × 1000 (10.69), and (TLG/ADCmean) × 1000 (57.53) predicted no evidence of disease; however, SUVmax (14.97), MTV (15.44), and TLG (83.07) predicted treatment failure. The patient showed no evidence of disease until 41 months follow-up after tonsillar cancer surgery

In the multivariate analyses, MTV, MTV/ADCmean, and TLG/ADCmean were each evaluated with significant clinicopathologic parameters (lymphatic invasion and perineural invasion). MTV (hazard ratio (HR) = 3.06, *P* = 0.010), MTV/ADCmean (HR = 3.12, *P* = 0.011), and TLG/ADCmean (HR = 4.33, *P* = 0.002) demonstrated significant results with lymphatic invasion (*P* = 0.026, 0.026, and 0.044, respectively) (Table 5 and Figs. 3 and 4).

**Table 5:** Multivariate analysis with significant clinicopathologic and ^18^F-PET/MRI parameters

Parameters	Model with MTV		Model with MTV/ADCmean		Model with TLG/ADCmean	
	Hazards ratio (95% CI)	*P* value	Hazards ratio (95% CI)	*P* value	Hazards ratio (95% CI)	*P* value
Lymphatic invasion (yes vs. no)	2.66 (1.13–6.27)	0.026*	2.66 (1.13–6.27)	0.026*	2.57 (1.02–6.45)	0.044*
Perineural invasion (yes vs. no)		NS				NS
MTV (> 11.08 vs. ≤ 11.08)	3.06 (1.31–7.13)	0.010*		N/A		N/A
(MTV/ADCmean) × 1000 (> 10.8 vs. ≤ 10.8)		N/A	3.12 (1.31–7.48)	0.011*		N/A
(TLG/ADCmean) × 1000 (> 108.9 vs. ≤ 108.9)		N/A		N/A	4.33 (1.72–10.87)	0.002*

*NS* not significant, *N/A* not assessed

*Statistically significant (*P* < 0.05)

## Discussion

This study demonstrated that combined PET/MRI parameters using ratio between metabolo-volumetric parameters and ADCmean can be independent prognostic factors for prediction of treatment failure in surgically resected head and neck cancer. The main advantage of our study is that we directly compared several metabolic and metabolo-volumetric parameters of PET, ADCmean of MRI, and combined PET/MRI parameters.

Among the PET parameters, SUVmax is the most commonly used parameter for quantitative analysis among the ^18^F-FDG PET/CT parameters, and SUVmax showed significant prognostic value in head and neck cancer [19]. In another study, SUVmean was suggested as a significant prognostic factor in head and neck cancer [20]. However, evaluation using metabolic parameters remains controversial, as tumor heterogeneity, the partial volume effect, time of SUV evaluation, measurement method, and body size may hinder the exact assessment of tumor characteristics [21]. In recent years, metabolo-volumetric parameters (MTV and TLG) have been considered more effective than metabolic parameters because tumor burden is considered by using the metabolo-volumetric parameters [22]. Previous direct comparison studies using metabolic and metabolo-volumetric parameters insisted that metabolo-volumetric parameters are superior to metabolic parameters in the prediction of head and neck cancer [23, 24]. In addition, ADC of MRI parameters also has prognostic value and reflects tumor cellularity. A previous study reported that ADC was a good prognostic factor for DFS in nasopharyngeal cancer [25]. In another direct comparison study with ^18^F-FDG PET/CT, ADC showed a potential to predict DFS in head and neck cancer similar to that of SUVmax [26].

PET parameters and ADC of MRI have complementary values. A study using SUV of ^18^F-FDG PET/CT and ADC of MRI demonstrated prognostic value in the head and neck squamous cell carcinoma separately and showed better risk stratification by combining SUV and ADC parameters [27]. However, as the modalities were different and the measurement of SUV and ADC was performed in a different manner, this study could not exactly show the direct comparison results. Recently, simultaneous ^18^F-FDG PET/MRI has been studied for head and neck cancer. A study showed that SUV and ADC of simultaneous ^18^F-FDG PET/MRI yield excellent results for detection of head and neck cancer recurrence [28]. This study showed the possibility of direct comparison between SUV and ADC parameters and their complementary values; however, metabolo-volumetric parameters of PET were not assessed. Another study using ^18^F-FDG PET/MRI revealed no significant correlation with metabolic parameters and ADC by direct comparison in head and neck cancer [29]. However, this study proved that each parameter was correlated with Ki-67 and nucleic area and that combined parameters (ratio between metabolic parameter and ADC) were correlated with nucleic area. We adopted this combined parameters method (named combined PET/MRI parameters (ratio between PET parameters and ADC)) in our study and evaluated its prognostic value. However, drawing VOI for ADC evaluation was another problem due to the lack of anatomical information caused by the suppressed signal in many normal tissues in DWI [11]. We adopted a PET-assisted ADC method, which showed significant predictive value in breast cancer unlike conventional ADC methods [30]. We hypothesized that as our combined PET/MRI parameters using the ratio between metabolo-volumetric parameter and ADC reflects metabolic activity, tumor burden and cellularity, they could show better prognostic value than each PET or MRI parameter.

Among the many clinicopathologic parameters, lymphatic invasion (lymph vessel invasion) was selected as a significant prognostic parameter in our study. Tumor lymphangiogenesis is a major component of metastatic process [31], and lymphatic invasion is thought to be a first step in the development of lymph node metastasis [32]. Previous studies lymphatic invasion indicates risk of lymph node metastasis and recurrence, thereby contributing to prognosis, in head and neck cancer [32, 33] and colorectal cancer [34].

Our study has some limitations. First, the HPV status of the patients showed no significant results on prognosis, which maybe due to small number of known HPV status. As many previous studies insisted that HPV status is an important factor for prognosis [35] and could affect the SUV [36] and ADC [37] measurements, it needs to be further studied with combined PET/MRI parameters in the future. In addition, SUV threshold for metabolo-volumetric parameters (MTV and TLG) is not clearly defined yet [38]. Moreover, the contoured ADC maps may not be entirely representative of the tumor as we did not contour ADC maps separately. Lastly, the diversity of adjuvant treatments could have confounded the results. A large-scale prospective study on the prognostic value of simultaneous ^18^F-FDG PET/MRI should be performed to confirm our results.

## Conclusions

In conclusion, combined PET/MRI parameters (PET metabolo-volumetric parameters corrected by tumor cellularity) on simultaneous ^18^F-FDG PET/MRI could be a possible predictor of treatment failure in surgically resected head and neck cancer. In addition, MTV of PET and lymphatic invasion were other independent prognostic parameters. We expect that simultaneous ^18^F-FDG PET/MRI with our combined PET/MRI parameters could become a prognostic imaging modality in head and neck cancer.

## Additional file

Additional file 1: Table S1. Comparison of ^18^F-FDG PET/MRI parameters according to type of treatment failure. (DOCX 30 kb)

## Abbreviations

ADC: Apparent diffusion coefficient
CCRT: Concurrent chemoradiation therapy
CEA: Carcinoembryonic antigen
CT: Computed tomography
DFS: Disease-free survival
DWI: Diffusion-weighted imaging
FDG: Fluorodeoxyglucose
FoV: Field of view
HPV: Human papillomavirus
LSO: Lutetium oxyorthosilicate
MIP: Maximum intensity projection
MRI: Magnetic resonance imaging
MTV: Metabolic tumor volume
OSEM: Ordered-subsets expectation maximization
PET: Positron emission tomography
RT: Radiation therapy
SD: Standard deviation
SUV: Standardized uptake value
TE: Echo time
TLG: Total lesion glycolysis
TR: Repetition time
VOI: Volume-of-interest
