# Supplementary material for: Prognostic value of simultaneous 18F-FDG PET/MRI using a combination of metabolo-volumetric parameters and apparent diffusion coefficient in treated head and neck cancer
Source: EJNMMI Res. 2018 Jan 10;8:2. doi: 10.1186/s13550-018-0357-9 (PMC5762617; doi:10.1186/s13550-018-0357-9)
Supplement: Additional file 1: Table S1. — Comparison of 18F-FDG PET/MRI parameters according to type of treatment failure. (DOCX 30 kb) [file 13550_2018_357_MOESM1_ESM.docx]

**Table S1.** Comparison of ^18^F-FDG PET/MRI parameters according to type of treatment failure

| ^18^F-FDG PET/MRI Parameters | Loco-regional recurrence (n = 10) | Distant metastasis (n = 12) | *P-*value |
| --- | --- | --- | --- |
| PET parameters |  |  |  |
| SUVmax | 10.0 ± 3.9 | 9.5 ± 4.1 | 0.771 |
| MTV | 11.9 ± 16.6 | 14.8 ± 10.3 | 0.228 |
| TLG | 67.7 ± 102.3 | 75.3 ± 63.8 | 0.254 |
| MRI parameters |  |  |  |
| ADCmean | 866.7 ± 251.8 | 794.4 ± 221.2 | 0.722 |
| Combined PET/MRI parameters |  |  |  |
| (SUVmax/ADCmean)X1000 | 12.4 ± 5.5 | 13.1 ± 6.6 | 1.000 |
| (MTV/ADCmean)X1000 | 13.3 ± 18.8 | 21.6 ± 21.2 | 0.093 |
| (TLG/ADCmean)X1000 | 75.6 ± 117.0 | 104.0 ± 92.7 | 0.140 |

SUVmax = Maximum Standardized Uptake Value; MTV = Metabolic Tumor Volume; TLG = Total Lesion Glycolysis;

ADCmean = Mean Apparent Diffusion Coefficient

*Statistically significant (*P* < 0.05)
